# Supplementary material for: Population-scale long-read sequencing uncovers transposable elements associated with gene expression variation and adaptive signatures in Drosophila
Source: Nat Commun. 2022 Apr 12;13:1948. doi: 10.1038/s41467-022-29518-8 (PMC9005704; doi:10.1038/s41467-022-29518-8)
Supplement: Supplementary file 3 — Description of Additional Supplementary Files [file 41467_2022_29518_MOESM3_ESM.pdf]

Description of Additional Supplementary Files

Supplementary Data 1.
Summary information for the 32 D. melanogaster strains sequenced in this work. (XLSX 14KB).

Supplementary Data 2
DNA extraction and sequencing metrics for the 32 genomes sequenced in this work. (XLSX 28KB)

Supplementary Data 3
Results from the reference-guided scaffolding process and CUSCO score estimates. (XLSX 24KB).

Supplementary Data 4
Comparison of the pairwise identity of TE sequences in the annotated ISO-1 genome and in the same genome assembled using our pipeline with the long-reads avaiable in Solares et al (2018). (XLSX 99KB).

Supplementary Data 5
Summary information about the 165 consensus TE sequences in the MCTE library used for annotating the 32 genomes in this study. (XLSX 24KB).

Supplementary Data 6
Comparisons between the annotation of the reference genome in Flybase and the annotation in this work, and comparison of the annotations of the 13 genomes with the BDGP and MCTE libraries. (XLSX 25KB).

Supplementary Data 7
Summary results for the assembly of the genomes of five Drosophila species and for the BLAST searches of the three unknown consensus sequences (new families) in these five genomes. (XLSX 10KB).

Supplementary Data 8
Comparison of the TE annotations performed with REPET, TIDAL and TEMP. (XLSX 56KB).

Supplementary Data 9
General overview of TE annotation per genome. (XLSX 48KB).

Supplementary Data 10
Characteristics of the 28,947 TEs annotated in the 47 genomes analyzed in this work that were transferred to the reference genome. (XLSX 5.3MB).

Supplementary Data 11
Frequency classification of the TEs annotated in the 47 genomes analyzed in this work. (XLSX 43KB).

Supplementary Data 12
eQTL analyses. (XLSX 202KB).

Supplementary Data 13
Analysis of selection evidence in regions flanking TE insertions. (XLSX 33KB).

Supplementary Data 14
Detection of CNVs and other TE variants in the regions flanking the 19 TE insertions associated with signatures of selection. (XLSX 15KB).

Supplementary Data 15
Enrichment analysis for genes nearby the 107 TEs with evidence of selection. IDs of the 107 TEs can be found in Table S13. (XLSX 89KB).

Supplementary Data 16
Family enrichment and gene location analysis for the 107 TEs showing evidence of selection. (XLSX 23KB).
