# Supplementary material for: Population-scale long-read sequencing uncovers transposable elements associated with gene expression variation and adaptive signatures in Drosophila
Source: Nat Commun. 2022 Apr 12;13:1948. doi: 10.1038/s41467-022-29518-8 (PMC9005704; doi:10.1038/s41467-022-29518-8)
Supplement: Supplementary file 20 — Reporting Summary [file 41467_2022_29518_MOESM20_ESM.pdf]

nature portfolio

Corresponding author(s): Josefa Gonzalez

Last updated by author(s): 2022/02/25

Reporting Summary

Nature Portfolio wishes to improve the reproducibility of the work that we publish. This form provides structure for consistency and transparency in reporting. For further information on Nature Portfolio policies, see our Editorial Policies and the Editorial Policy Checklist.

Statistics

For all statistical analyses, confirm that the following items are present in the figure legend, table legend, main text, or Methods section.

n/a	Confirmed	
☐	☒	The exact sample size (*n*) for each experimental group/condition, given as a discrete number and unit of measurement
☐	☒	A statement on whether measurements were taken from distinct samples or whether the same sample was measured repeatedly
☐	☒	The statistical test(s) used AND whether they are one- or two-sided<br>*Only common tests should be described solely by name; describe more complex techniques in the Methods section.*
☐	☒	A description of all covariates tested
☐	☒	A description of any assumptions or corrections, such as tests of normality and adjustment for multiple comparisons
☐	☒	A full description of the statistical parameters including central tendency (e.g. means) or other basic estimates (e.g. regression coefficient) AND variation (e.g. standard deviation) or associated estimates of uncertainty (e.g. confidence intervals)
☐	☒	For null hypothesis testing, the test statistic (e.g. *F*, *t*, *r*) with confidence intervals, effect sizes, degrees of freedom and *P* value noted<br>*Give P values as exact values whenever suitable.*
☒	☐	For Bayesian analysis, information on the choice of priors and Markov chain Monte Carlo settings
☐	☒	For hierarchical and complex designs, identification of the appropriate level for tests and full reporting of outcomes
☐	☒	Estimates of effect sizes (e.g. Cohen's *d*, Pearson's *r*), indicating how they were calculated

Our web collection on statistics for biologists contains articles on many of the points above.

Software and code

Policy information about availability of computer code

Data collection	No software was used
Data analysis	Albacore Sequencing Pipeline Software (v.2.2); NanoPlot (v.1.19); Canu (v.1.7); FinisherSC (v.2.1) ; SMRT Link v.5.0.1; Racon (v.1.0); Nanopolish (v.0.10.1); Pilon (v.1.22) ; GATK (v.4.0) ; bcftools stats (v.1.9) ; purge_haplotigs (v.1.0.1) ; MUMmer (v.4.0); RaGOO (v.1.02) ; minimap2 (v.2.9) ; MUMmer4 (v.4.0); BUSCO (v.3.0.2) ;MAFFT v.7.4 ; REPET package (v.2.5); PASTEC (v2.0) ; BLAT (v.35) ; RepeatMasker (v.3 and v.4) ; MUSCLE (v.3.5) ; Geneious (v.10.0.2) ; TEMP (v.1.05) ; TIDAL (v.1.0) ; BEDtools (v.2.18); minimap2 (v.2.9) ; UpSetR (v.1.3) ; VennDiagram (v.1.6) ; fastp package (v.0.20) ; salmon package (v.1.0.0) ; DESeq2 (v.1.28.1) ; QTLTools package (v.1.2) ; selscan (v.1.2.0a) ; GATK (v.4.0) ; SHAPEIT4 (v.4.1) ; DAVID (v.6.8) . https://github.com/gabyrech/deNovoTEsDmel; https://github.com/sradiouy/deNovoTEsDmel

For manuscripts utilizing custom algorithms or software that are central to the research but not yet described in published literature, software must be made available to editors and reviewers. We strongly encourage code deposition in a community repository (e.g. GitHub). See the Nature Portfolio guidelines for submitting code & software for further information.

Data

Policy information about availability of data

All manuscripts must include a data availability statement. This statement should provide the following information, where applicable:

- Accession codes, unique identifiers, or web links for publicly available datasets
- A description of any restrictions on data availability
- For clinical datasets or third party data, please ensure that the statement adheres to our policy

All the databases/datasets used in the study along with appropriately accessible links/accession codes are included in the manuscript under the "data availability" section as well as in this reporting summary. For example: all scaffolded assemblies and the raw data (long and short read sequencing) have been deposited in NCBI

under the BioProject accession PRJNA559813. The VCF file containing SNP callings for 46 D. melanogaster genomes used for testing positive selection evidences is available at http://dx.doi.org/10.20350/digitalCSIC/13708. Fasta sequences for the D. melanogaster Manually Curated Transposable Elements (MCTE) library are available at http://dx.doi.org/10.20350/digitalCSIC/13765. The new consensus sequences were deposited in Dfam (Storer et al 2021). Recombination rates according to Fiston-Lavier et al. (2010) and Comeron et al. (2012) for D. melanogaster genome release 6 are available at http://dx.doi.org/10.20350/digitalCSIC/13766. BED files containing Transposable Element (TE) annotations for 47 Drosophila melanogaster genomes are available at: http://dx.doi.org/10.20350/digitalCSIC/13894.

Field-specific reporting

Please select the one below that is the best fit for your research. If you are not sure, read the appropriate sections before making your selection.

[x] Life sciences [ ] Behavioural & social sciences [ ] Ecological, evolutionary & environmental sciences

For a reference copy of the document with all sections, see nature.com/documents/nr-reporting-summary-flat.pdf

Life sciences study design

All studies must disclose on these points even when the disclosure is negative.

Sample size	32 genomes that represent European climates and one North American population were sequenced in this work. These genomes were chosen to represent the diversity of climatic regions present in Europe.
Data exclusions	No data were excluded from the analysis
Replication	No replication of the sequencing genomes was performed. This is the standard in the field.
Randomization	No experimental groups
Blinding	No group allocation

Reporting for specific materials, systems and methods

We require information from authors about some types of materials, experimental systems and methods used in many studies. Here, indicate whether each material, system or method listed is relevant to your study. If you are not sure if a list item applies to your research, read the appropriate section before selecting a response.

Materials & experimental systems

n/a	Involved in the study	
[x]	[ ]	Antibodies
[x]	[ ]	Eukaryotic cell lines
[x]	[ ]	Palaeontology and archaeology
[ ]	[x]	Animals and other organisms
[x]	[ ]	Human research participants
[x]	[ ]	Clinical data
[x]	[ ]	Dual use research of concern

Methods

n/a	Involved in the study	
[x]	[ ]	ChIP-seq
[x]	[ ]	Flow cytometry
[x]	[ ]	MRI-based neuroimaging

Animals and other organisms

Policy information about studies involving animals; ARRIVE guidelines recommended for reporting animal research

Laboratory animals	Drosophila melanogaster natural strains from Europe and North America
Wild animals	5-10 day old Drosophila melanogaster female flies
Field-collected samples	Female flies were collected in the field and inbred lines were established in the lab
Ethics oversight	No ethical aproval was needed

Note that full information on the approval of the study protocol must also be provided in the manuscript.
